# 

**Published:** 1876-11

**Authors:** 


[Vol. XVI] NOVEMBER, 1876. [No. 4.]

### BUFFALO

### Medical and Surgical Journal.

S.G.O.L. INDEXED

EDITED BY EDWARD N. BRUSH, M. D.

BUFFALO.
PUBLISHED FOR THE EDITOR.

1876.

